# A Simulation Tool for Dynamic Contrast Enhanced MRI

**DOI:** 10.1371/journal.pone.0057636

**Published:** 2013-03-14

**Authors:** Nicolas Adrien Pannetier, Clément Stéphan Debacker, Franck Mauconduit, Thomas Christen, Emmanuel Luc Barbier

**Affiliations:** 1 Institut National de la Santé et de la Recherche Médicale, U836, Grenoble, France; 2 Université Joseph Fourier, Grenoble Institut des Neurosciences, Grenoble, France; 3 Stanford University, Department of Radiology, Stanford, California, United States of America; University of California Davis, United States of America

## Abstract

The quantification of bolus-tracking MRI techniques remains challenging. The acquisition usually relies on one contrast and the analysis on a simplified model of the various phenomena that arise within a voxel, leading to inaccurate perfusion estimates. To evaluate how simplifications in the interstitial model impact perfusion estimates, we propose a numerical tool to simulate the MR signal provided by a dynamic contrast enhanced (DCE) MRI experiment. Our model encompasses the intrinsic 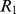 and 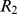 relaxations, the magnetic field perturbations induced by susceptibility interfaces (vessels and cells), the diffusion of the water protons, the blood flow, the permeability of the vessel wall to the the contrast agent (CA) and the constrained diffusion of the CA within the voxel. The blood compartment is modeled as a uniform compartment. The different blocks of the simulation are validated and compared to classical models. The impact of the CA diffusivity on the permeability and blood volume estimates is evaluated. Simulations demonstrate that the CA diffusivity slightly impacts the permeability estimates (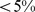 for classical blood flow and CA diffusion). The effect of long echo times is investigated. Simulations show that DCE-MRI performed with an echo time 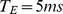 may already lead to significant underestimation of the blood volume (up to 30% lower for brain tumor permeability values). The potential and the versatility of the proposed implementation are evaluated by running the simulation with realistic vascular geometry obtained from two photons microscopy and with impermeable cells in the extravascular environment. In conclusion, the proposed simulation tool describes DCE-MRI experiments and may be used to evaluate and optimize acquisition and processing strategies.

## Introduction

Bolus-tracking MRI techniques are widely used in clinical and preclinical studies to obtain imaging biomarkers that predict tumors progression and outcome [1], [2]. Depending on the predominant contrast in use, two different techniques can be employed: 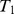-weighted dynamic contrast enhanced MRI (DCE-MRI) [3] or 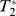-weighted dynamic contrast susceptibility (DSC-MRI) [4]. DSC-MRI is the approach of choice for measuring perfusion biomarker in the brain. DCE-MRI is preferred in other organs [5], [6] where the contrast agent (CA) leaks outside of the vessels. It is also used to assess the vessel permeability in the brain when the blood brain barrier (BBB) is disrupted. These techniques emerged at the same time about 20 years ago but their quantification remains challenging.

In a brain voxel with intact BBB, the CA yields a transient, strong increase in voxel 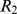 and 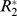 due to the increase in the magnetic susceptibility difference (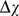) between blood and tissue, and a more subtle increase in voxel 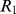 due to the increase in blood 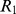 and the water exchange between intra and extravascular compartments [7]. In a tissue with an altered BBB, the CA leaks across the vessels and 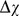 is reduced at the vessel wall, limiting the increase in voxel 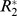 whereas the 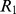 effect is enhanced. Additionally, the distribution of CA around extravascular impermeable cells further perturbs the magnetic field and increases 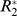 which then competes with the 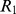 enhancement. The intricacy of these phenomena makes the MR signal interpretation arduous.

In DCE-MRI, the analysis is made with compartment models which handle blood flow and CA exchanges but often lack methods to deal with the NMR signal. Ideally, one would combine the compartment models which describe the microscopic 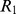 and 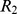 changes [8]–[11] with a model that describes the perturbations of the magnetic field induced by the susceptibility interfaces [12]–[15]. Recent progress to untangle these phenomena have been made by measuring DSC-MRI and DCE-MRI simultaneously using multi-echo sequences [16], [17]. The analysis of these acquisitions requires the use of advanced analytical models [18] that can potentially provide new biomarkers [19]. However, the impact of the CA diffusion within the extravascular space on the MR signal is disregarded and the effect of the arising susceptibility gradients around the cells remains unclear. A better description of the entanglement of these various effects within a voxel is thus of considerable interest.

In this article we report a numerical model of the MR signal in a DCE-MRI like experiment acquired with a multi gradient-echo sequence over several minutes. Within an affordable computing time, the proposed approach considered: the effect of the magnetic field perturbations caused by vessel and cell interfaces, the diffusion of water molecules, the blood flow, the CA leakage across the vessel wall and the CA diffusion within the extravascular space. The CA distribution is considered uniform within the vascular compartment (plug-flow or not well mixed compartment are not considered). The algorithm relies on:

1. A compartment model to simulate the CA exchange between the capillary bed and the tissue.
2. The computation of the magnetic field induced by the susceptibility variations using a Fourier based approach [20], [21].
3. The deterministic approach introduced by Bandetini et al. [22] and further developed by Klassen et al [23] to model the MR signal provided by free diffusing water molecules within an inhomogeneous magnetic field.

The impermeable vascular and cellular membranes were handled by a modified convolution kernel approach. The model was first validated and compared to simple cases where analytical solutions exist. The impact of the CA diffusion on the permeability estimate was then investigated and the flexibility of the tool was eventually demonstrated by running the simulation on vascular networks obtained from optical microscopy and on geometries with extravascular cells.

## Methods

### Algorithm

Simulations were performed in the Matlab environment (Mathworks Inc. Natick, MA, USA) on a Dell Precision computer (double quad 2.33 GHz Intel Xeon processor, RAM 32 GB) and at the CNRS/IN2P3 Computing Center (Lyon/Villeurbanne - France). The step time was set to 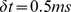 and the total duration of the simulated DCE experiment was 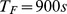. To simulate a 900s-long DCE experiment (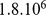 frames given our 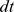) with moderate computation time, simulations were performed in 2D. The overall time was about 14 days for a single DCE experiment. In the following, bold capital letters refer to 2D-lattices. II refers to the lattice filled with 1, 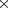 to the point wise multiplication, 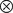 to the convolution and 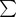 to the 2D summation over a lattice. Summation of a lattice and a scalar is done point wise.

**Figure 1:**
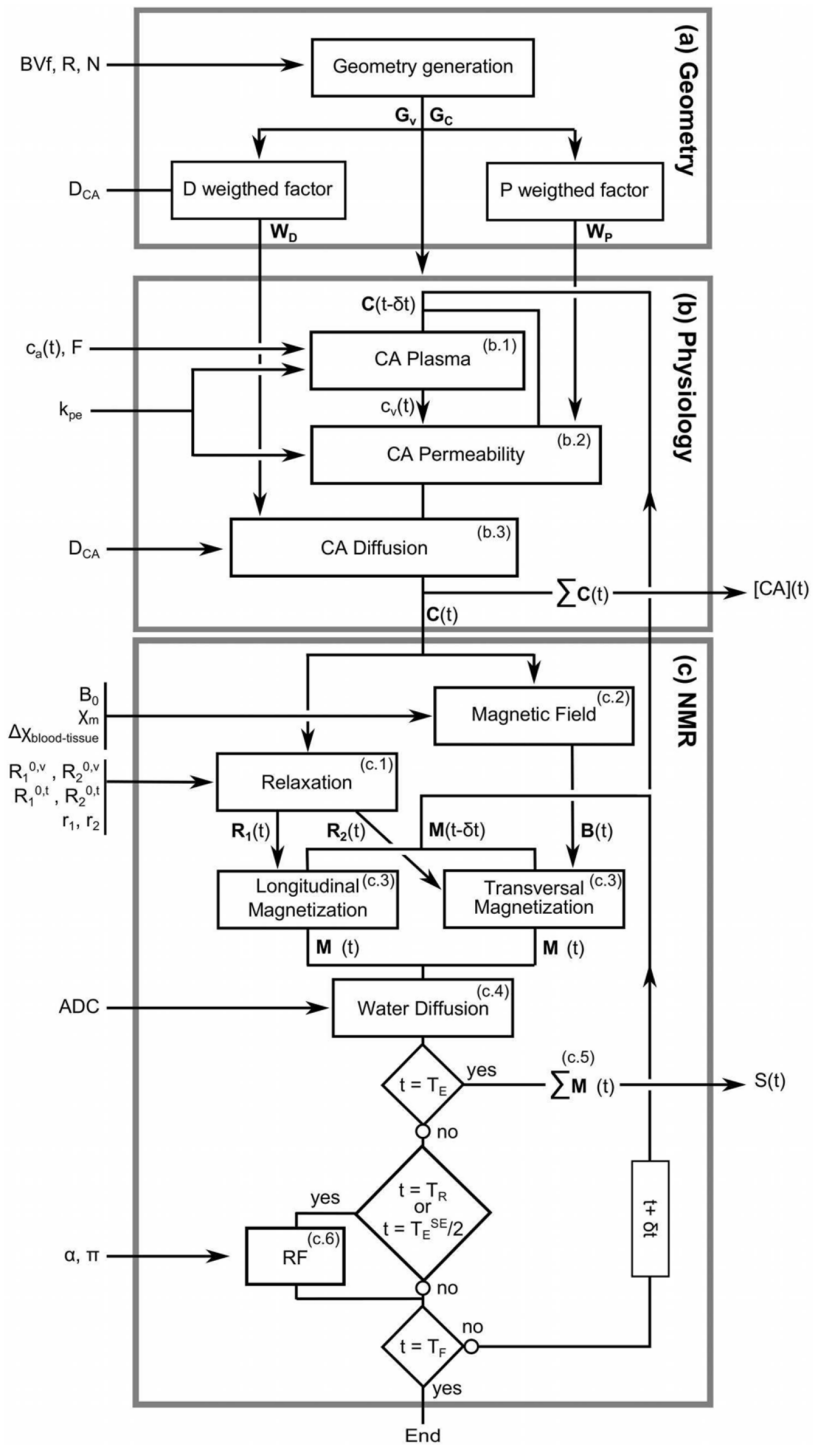
Algorithm sketch of the simulation. Only the most important parameters have been represented. Data on the left of the gray boxes are inputs to the model. Data on the right are outputs of the simulation. The simulation is organized in three blocks. Block (a) initializes the geometry. Block (b) describes the CA behavior over time. Block (c) estimates the MR signal.

The simulation tool was organized in three distinct blocks: Geometry, Physiology and NMR (Fig. 1, Table 1).

**Table 1:** List of the main parameters used in the algorithm.

Name	Definition	Units
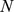	Number of vessels	-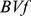	Blood Volume fraction	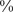
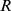	Radius of the vessel	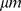
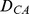	Diffusivity of the CA	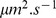
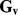	Vessel lattice	-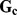	Cells lattice	-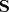	Contact surface lattice	-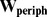	Permeability weighting lattice	-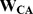	Diffusion weighting lattice	-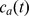	Arterial input function	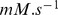
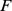	Blood flow	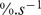
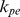	Permeability rate	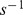
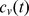	Plasma CA concentration	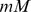
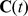	CA concentration lattice	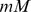
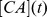	Mean CA concentration in 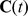	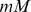
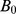	Static magnetic field	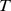
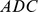	Apparent Diffusivity of the water	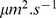
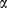	RF flip angle	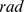
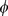	RF phase angle	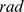
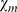	Molar magnetic susceptibility of the CA	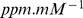
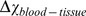	Original Magnetic susceptibility between blood and tissue	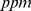
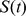	MR signal	-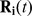	Relaxation rates lattices	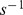
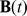	Magnetic field lattice	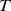
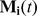	Magnetization lattices	-

#### (a) Geometry block

The geometry was designed on a 2D plane sampled with 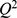 pixels. 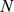 vessels with radius 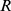 were randomly spread out orthogonal to the plane under periodic boundary condition, defining the blood surface fraction 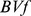 (equivalent to a volume fraction in 3D).

Cells were spread out with the same boundary condition and occupy a surface defined by the porosity 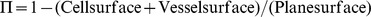. The cell radius was initially set to 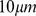 and slowly shrunk to obtain the desired porosity.


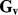
 denoted the lattice with 1 inside the vessels and 0 outside. 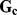 denoted the lattice with 1 inside the cells and 0 outside.

#### (b) Physiology block

This block modeled the CA concentration, 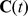, within the plane.

*(b.1) Plasma.* The time evolution of the concentration of CA in the vessels, 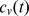, was described by the discretized form of a two compartments model where the CA enters the tissue via the arterial influx, leaves by venous outflux and exchange by transendothelial leakage. No spatial variation of CA caused by plug-flow or not well-mixed space are considered.
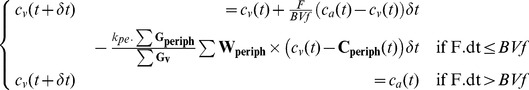
(1)where 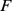 denotes the blood flow in surface fraction per second (equivalent to a volume fraction in 3D), 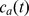 the arterial input function (AIF) and the terms modeling for the permeability are defined in the following paragraph. 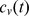 was considered the same in each vessel. The AIF was an input to the physiology block.

**Figure 2:**
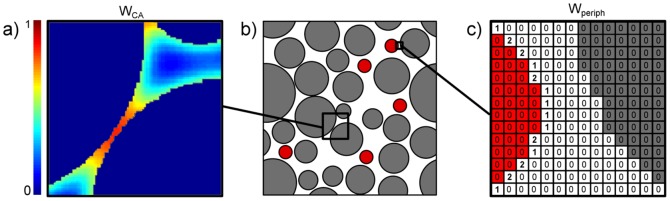
Illustration of the weighting lattices 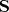 and 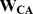. (a) Zoom in the diffusion weighting lattice 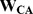. The diffusion appears restricted near the membranes. (b) Illustration of the geometry lattices. In red, the vessel, in grey the cells. (c) Zoom in the surface weighting lattice 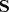 that computes the number of contact exchange interfaces between a vessel and its periphery.

*(b.2) Permeability*. The CA exchanges between the vessels and the extravascular space occurred only at the periphery of each vessel. 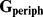 was defined as the lattice with 1 in the one-pixel wide periphery of each vessel (connectivity 4) and with 0 outside. The CA concentration lattice in that region was denoted 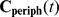. At each time step, we considered the CA exchange between the vessels and its periphery. The amount of CA that extravasates was modeled by a first order kinetic law with exchange rate 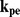. The same exchange rate was considered in both ways:
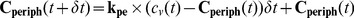
(2)In 3D, one generally defines 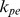 as the exchange rate between the vessel and the extravascular extracellular volume, 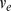 (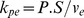, with 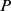 the permeability and 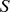 the surface exchange). In our 2D approach, we must consider the volume in which the CA extravasates, which is reduced to the surface 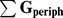, plus the contact exchange which is not equivalent for every points in the periphery of the vessels. Thereby, to remain consistent with the literature, 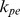 was scaled by 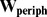:
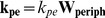
(3)with
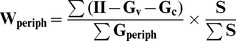
(4)where the first fraction accounts for the volume scaling and the second for the differences in the contact exchange. 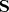 was computed as (see also Fig. 2C for an illustration of 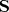):
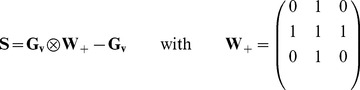
(5)


The concentration lattice 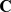 was eventually updated with 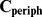:
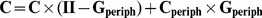
(6)


*(b.3) CA Diffusion*. The diffusion of CA into the extravascular space was modeled with a Gaussian diffusion kernel, denoted 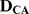, as described by Eq.[7]:
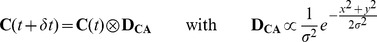
(7)where the mean square displacement of a CA molecule is 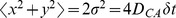 (in 2D, [22]) and 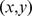 the coordinates in the plane. The kernel 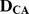 was designed with the same size as 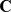.

The diffusion of CA described by Eq.[7] should neither contribute to the transendothelial transport modeled by Eq.[2] nor diffuse within the cells. We thus introduced a bounce-like mechanism for CA transport at the membranes of the vessels and cells characterized by the following weighting lattice (see also illustration in Fig. 2A):
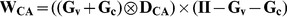
(8)For each point of the lattice, 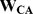 defines the amount of CA that would have diffused from one point into the cells or vessels in the case of free diffusion. At each time step, this amount has to be sent back to the extravascular extracellular space. In our case, it is sent back to where it originates. The evolution of 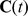 was thus computed with Eq.[9]:
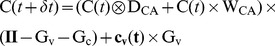
(9)


Special attention was paid to the kernel width to minimize physically impossible behaviors of CA such as jump over obstacles. The step time 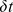 was set according to 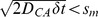 where 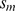 was the characteristic size of the smallest obstacles (see discussion section for further details). Each step that involved a convolution was computed using the FFT algorithm. The mean CA concentration in the plane, 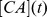, was computed by averaging 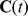.

#### (c) NMR block

This block modeled the magnetiztion, 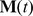, within the plane.

*(c.1) Relaxation*. Longitudinal and transverse relaxation lattices were calculated from 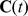 based on Eq.[10]:
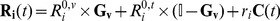
(10)where index i stands for 1 or 2, 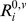 and 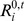 are the initial relaxation rates of the vascular and tissue compartments respectively and 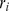 is the CA relaxivity.

*(c.2) Magnetic field*. The perturbations of the magnetic field induced by the susceptibility variations in the plane were computed using a Fourier based approach [20], [21] adapted here in 2D, Eq.[11]:
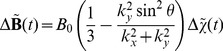
(11)where 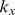 and 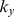 are the coordinates in the Fourier space, 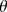 the angle between the normal to the plane and 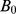 and the tilde, 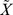, denotes the Fourier transform of X. The susceptibility map was defined by 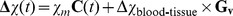 where 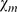 is the molar magnetic susceptibility of the CA and 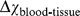 the original magnetic susceptibility difference between the vessel and tissue. The perturbation of the magnetic field was averaged over 3 orthogonal orientations of the plane with respect to 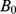 to mimic an isotropic distribution of vessels in a 3D voxel (see validation in results section).

*(c.3) Magnetization*. The magnetization evolution was described by the Bloch equations, Eq.[12]:
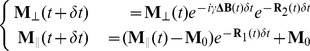
(12)with 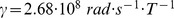 and 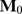 the longitudinal magnetization at equilibrium. The symbol 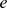 denotes here a point wise exponentiation.

*(c.4) Water diffusion*. The diffusion of water was modeled by applying a diffusion kernel, 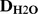, on the magnetization lattices as already proposed [22], [23]:
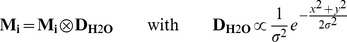
(13)where the mean square displacement of a water molecule is 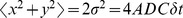 (in 2D, [22]) and 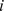 stands for 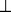 or 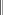. The convolution with the kernel modeled the probability of the spins to move to a different location during the time 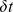. Due to the finite extension of the lattice, the kernel was subsequently normalized to unity. We assumed that water diffused freely within the plane and between compartments. The convolution was performed using the FFT algorithm.

*(c.5) MR sequence*. A saturation-recovery sequence with a multi gradient-echo acquisition scheme was simulated. At each echo time 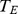, the MR signal was sampled by summing the transverse magnetization 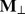 across the lattice. At 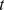 modulo 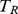, RF excitation pulse, characterized by flip angle 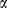 and phase 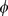, was applied on the magnetization lattices. For spin-echo case, a 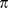 RF pulse was applied at 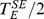.

Unless mentioned otherwise, the parameter values used in the simulation were as follows. The static magnetic field 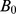 was set to 4.7T, the time step 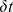 to 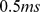 and the total duration of the experiment 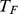 to 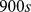. The geometry modeled was a 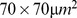 plane sampled with 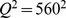 elements (lattice point size 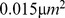). 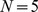 vessels of radius 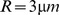 were generated, filling up 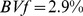. These values match in vivo measurements in healthy tissue [24], [25]. In vivo measurements made in rodents provided the AIF shape, 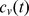 [26]. Relaxation properties of the CA matched Gd-chelate ones: 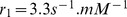, 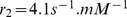 (data from Guerbet, France). The initial relaxation rates were, in the vessels, 
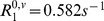
 [27], 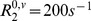 (higher than previously reported value [28]), in the tissue, 
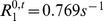
 [29], 
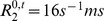
. We used 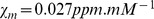 for the molar susceptibility of the CA [30] and 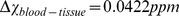 (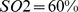 and 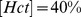 at 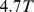 [31]) (CGS units). The water diffusion was set to 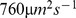 [32]. 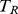 was set to 500 ms, 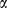 to 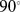 and 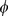 to 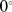 [26].

**Figure 3:**
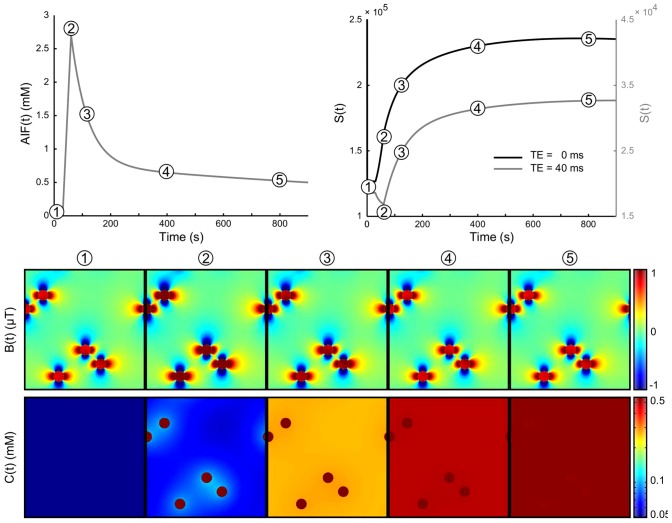
Illustration of the evolution of the concentration of CA. CA concentration in the vessels 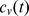 (a) and the corresponding MR signal 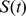 (b). 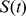 is simulated for 2 echo times: 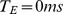 (black) and 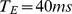 (grey). The change in CA concentration 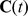, represented by the lattices, and in the magnetic field perturbations 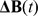 are presented at five times points labeled (1) to (5). For this longer echo time, one can observe the competition between the susceptibility effect which decreases the signal (point (2)) and the enhancement produced by the 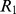 relaxation effect of the CA which extravasates into the tissue (points (3) to (5)). At the last simulation time point (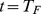) (5), 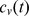 is lower than 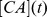 (not shown) and the concentration in the extravascular space begins to decrease. Note the log scale for 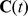 introduced for sake of clarity.

As a reference for 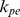, we used 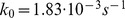 and 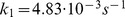, values reported in [26] for healthy muscle tissue and for tumor tissue. An illustration of the shape of the AIF is presented in Fig. 3b. For 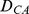, we used 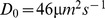 which correspond to the coefficient of diffusion for Gd-DOTA measured in rat brain [33]. The free diffusion of Gd-DOTA, 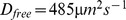, has also been reported [33].

### Validation

#### Contrast Agent Diffusion

To evaluate our kernel based approach used to model constrained diffusion, we compared our results to a 2D Monte-Carlo (MC) simulation. In the MC approach, elastic rebounds of CA on the surface of obstacles were considered [34]. Diffusion with both approaches was simulated within the same geometry, 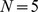 and 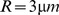. The apparent diffusion coefficient of Gd was set to 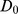. Due to the extensive computation time of the MC approach, we simulated the diffusion process during 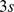. The CA was initially positioned at the center of the grid: 1 mM in a single pixel for the kernel simulations, 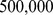 particles randomly spread within the same pixel for the MC simulations. To allow comparison between the two approaches, the total amount of matter of each approach was equalized afterwards.

#### Blood flow and CA Permeability

To investigate the validity of the approach used to model the permeability, we fitted the concentration profiles with the classic modified Tofts model [8] using a nonlinear Levenberg-Marquardt algorithm.
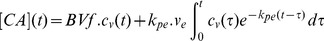
(14)We eventually compared the estimate of 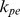, 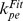, with the theoretical 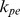, 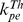, introduced as an input of the simulation at step (b.2). We denoted 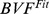 the estimate of 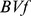.

#### Magnetic Field Perturbations

To validate the 2D technique used to compute the magnetic field perturbations, we balanced the corresponding 3D model (magnetic field perturbations induced by isotropic distributed cylinders in space and orientation [35]) with 3 different 2D approaches: 1 unique vessel in 1 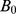 orientation, N vessels in 1 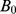 orientation, N vessels in 3 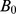 orientations. For each approach, we simulated and averaged the free induction decays (FID) provided by a set of 70, randomly obtained, geometries (only 1 geometry for the first 2D approach, and we estimated 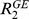 by a mono-exponential function fitted to the mean FID with a nonlinear Levenberg-Marquardt algorithm. Each geometry had the same properties: 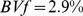, 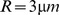, 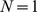 or 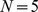 for the 2D approach, 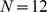 for the 3D approach. To match the conditions described by [35], the magnetic susceptibility difference between the vessels and the tissue was set to 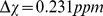.

#### Relaxation changes vs vessel radius

To further investigate the validity of the proposed approach, we simulated the dependency of the gradient-echo (GE) and spin-echo (SE) relaxation rates (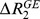 and 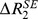) with the radius of the vessels as previously described [36]–[38]. For a given vessel radius, we randomly generated a set of 10 different geometries with 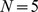 vessels that occupy 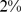 of a plane. To match these constraints, the plane size was adjusted to the vessel radius. Eighteen vessel radii were simulated, between 1 and 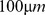. To match the conditions used by Boxerman et al. [37], the diffusion of water was set to 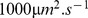, 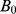 to 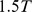 and 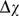 to 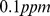. The MR signal was simulated at 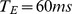 for gradient-echo type experiment and at 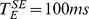 for spin-echo type experiment. The relaxation rates were computed with Eq.[15]:
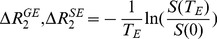
(15)


### Impact of Diffusion, Permeability and Echo Time

The concentration 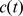 was derived from the MR signal 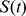 as described in [26]:
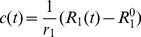
(16)with
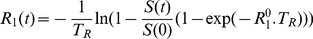
(17)


This equation is valid only under certain assumptions (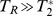, 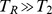 (no stimulated echoes), 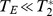, 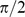 flip angle, no inflow, etc.). We then fitted either 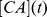 or 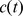 with the bi-compartment model (Eq.[14]) to derive the errors on estimated permeability constant: 
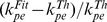
.

## Results

For sake of simplicity, unless mentioned otherwise, the results presented in this study are obtained with *no cells positioned in the extravascular space* and *in the limit of high flow* (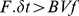), i.e. 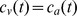.

As an illustration, Fig. 3 shows the changes in 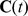 and 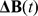 throughout the simulation together with the input 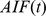 and the output 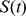 at two gradient-echo times: 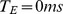 and 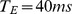, with 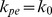 and 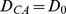.

### Validation

#### Contrast Agent Diffusion

**Figure 4:**
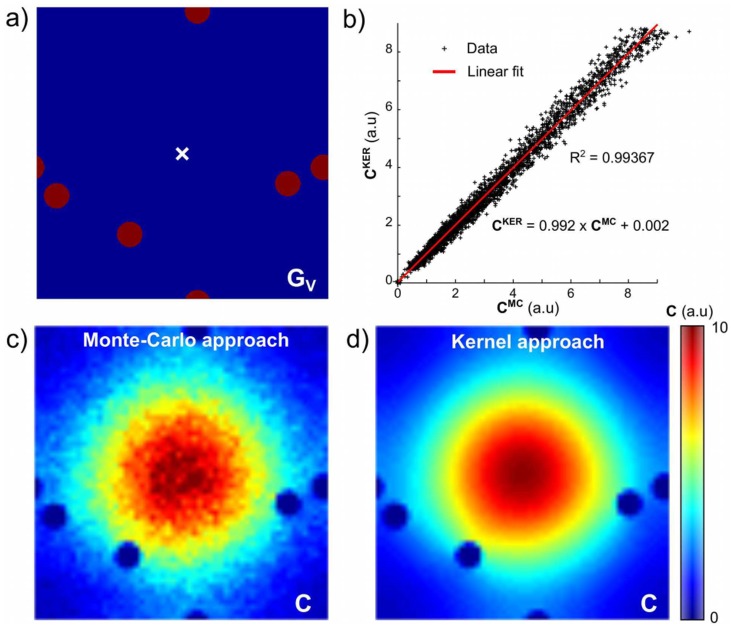
Comparison between MC approach and kernel based approach for modeling the CA diffusion. (a) Geometry used, 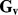. The white cross indicates where the CA was initially placed. (b) Spatial correlation plot between 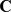 obtained via the convolution with a diffusion kernel and 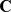 obtained with the MC approach after normalization. (c–d) Final maps of CA concentration, 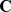, for the MC approach (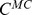) and the kernel approach (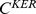), respectively (smoothed and undersampled to a 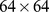 lattice).

Fig. 4a presents the geometry lattice 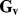 and the injection site. Fig. 4c and Fig. 4d show the diffusion maps obtained with the MC and the kernel approaches respectively. Due to low SNR for the MC approach, the displayed lattices were smoothed to ease their visualization. Fig. 4b shows the correlation graph between the two approaches. The two maps are in good agreement (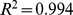) with a slightly faster diffusion process observed in the MC approach (slope = 0.992).

#### Blood flow and CA Permeability

**Figure 5:**
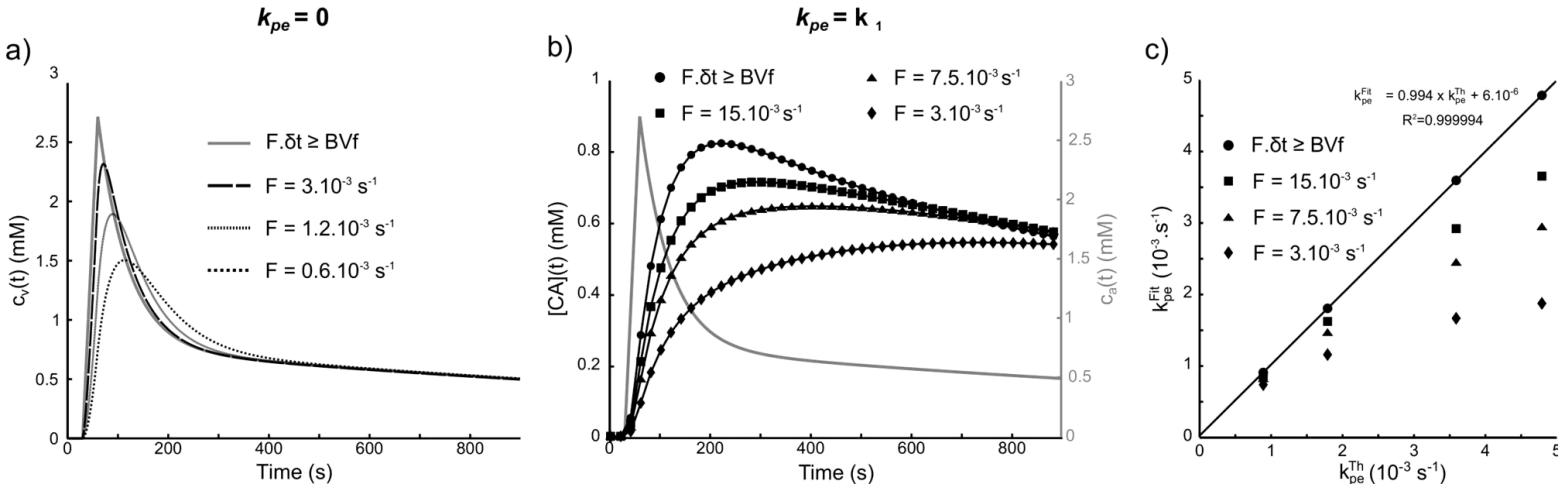
Concentration profiles for various blood flows and permeabilities to CA. (a) Concentration of CA in the vascular compartment, 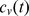, as a function of time for impermeable vessel wall (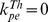) and different blood flow values. (b) Time course of 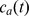 and 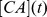 for 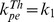 and different blood flow values. 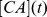 is plotted every 20s to ease readability. The plain black lines represent the fit obtained with the Tofts model (Eq.[14]). Note the difference in scale for the arterial input function, 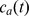. (c) Plots of the estimated permeability coefficient 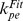 and the input value 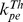 for different blood flows and permeabilities to CA. A linear fit is obtained in the case of high flow (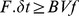). For lower blood flows, the model failed to distinguish the flow from the permeability and 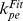 is underestimated.

The results of this section were obtained with 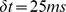. Fig. 5a shows the concentration profiles obtained for different blood flow values when 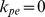. When 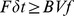, the flow is high enough to renew the blood volume at each time step 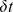 and 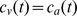. When 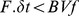, i.e 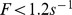 in our experimental conditions, the concentration profiles are modified accordingly to the dilution of the arterial input within the vascular compartment. This situation corresponds to a single compartment model with a mono-exponential residue function with characteristic time 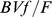.

Fig. 5b illustrates the time courses of 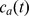 and 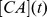 when 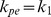 for different blood flows 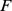: 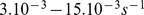 (equivalent to 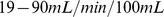). 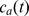 is an input to the simulation (step b.1) and 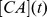 is the output of block b. As depicted by the concentration curve shapes 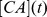, the uptake of CA is limited when the blood flow decreases.

We simulated the change in 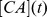 for 4 permeability values, 4 blood flows and with 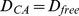 (to mimic an infinite diffusion coefficient as assumed by a bi-compartmental model). The Tofts model properly fits the data for the range of permeabilities and blood flow simulated (Fig. 5b). Fig. 5c presents the results of the estimated parameter 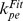. For high flow, a linear correlation between 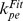 and 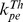 is observed (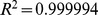). The slope is slightly lower than 1 and demonstrates that 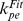 underevaluates 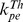 when the CA diffusion is slightly constrained (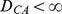). As already reported, for lower blood flow values, the CA leakage is limited by the inlet and the model fails to distinguish the blood flow from the permeability [8].

#### Magnetic Field Perturbations

**Figure 6:**
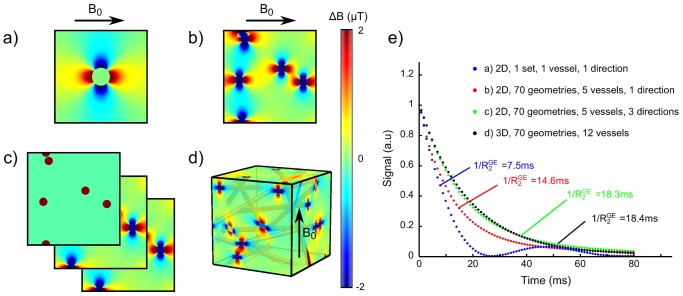
Impact of various magnetic field computations on the FID simulation. (a) 1 vessel in 1 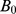 orientation (b) N vessels in 1 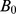 orientation (c) N vessels in 3 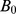 orientations (d) N vessels in 3D. The vessel arrangement is presented in 3D and for display, the magnetic field perturbation is only presented on each face of the cube but is computed in 3D. (e) Normalized FID for approaches (a)–(d) (averaged across the geometries for approaches (b–d)).

The different voxel configurations are displayed on Fig. 6a–d. Fig. 6e presents the mean normalized FID obtained with each approach. The single vessel approach results in a very short decay (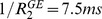) whereas the N vessels averaged over 3 directions approach (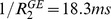) presents a decay similar to what is obtained with the 3D approach (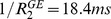).

#### Relaxation changes vs vessel radius

**Figure 7:**
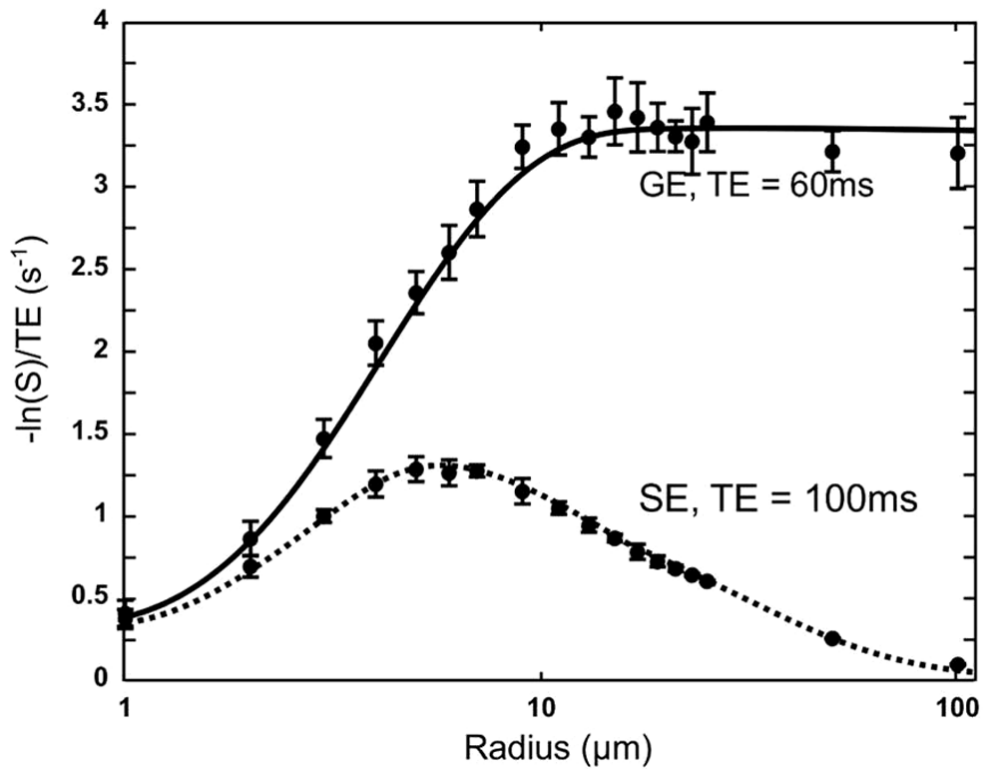
Vessel radius dependence of 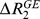 and 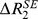. Parameters values are 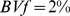, ADC = 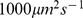, 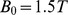 and 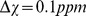. 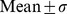 across 10 geometries. The data presented here are in excellent agreement with those reported in [37].

Fig. 7 shows the dependence of 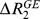 and 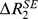 on the vessel radius. The results of our simulation are in very good agreement with similar works obtained with other approaches [37], [38]. We also observe that the standard deviation is larger for the GE experiments than for the SE experiments.

### Impact of Diffusion, Permeability and Echo Time

We simulated a DCE experiment with various CA diffusion coefficients (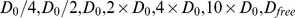), various permeabilities to CA (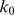, 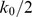, 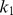) and various 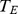 (20 echoes, 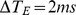).

**Figure 8:**
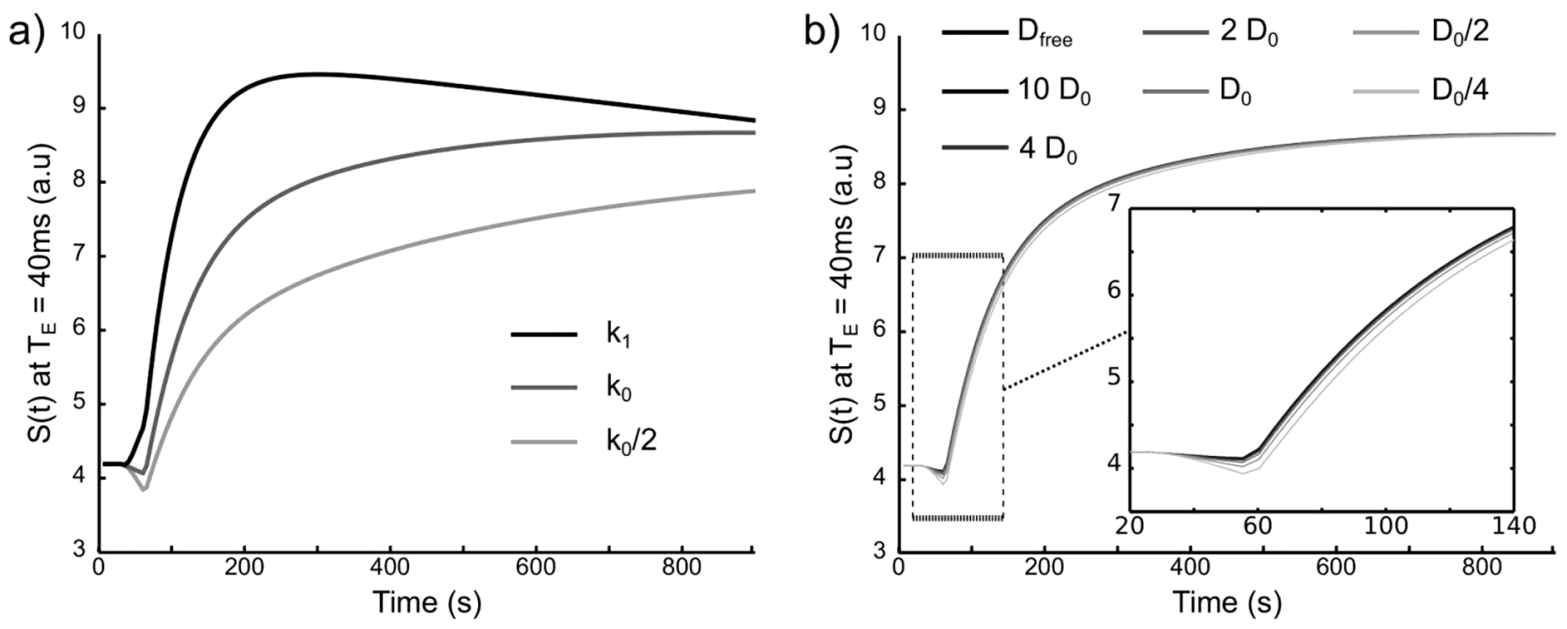
Change in the MR signal for different 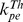 and 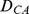 values. (a) S(t) at 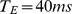 for 3 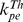 values: 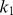, 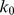 and 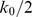 with 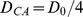. (b) S(t) at 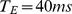 for 7 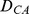 values: 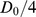, 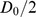, 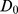, 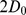, 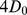, 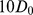 and 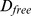 with 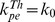.

Fig. 8 illustrates 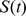 obtained for different 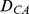 values and 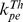 values. As expected, 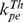 strongly impacts the shape of 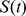 (Fig. 8a). Conversely, the CA diffusion coefficient has a small impact on the shape of 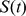. The highest deviation appears when the CA concentration in the vessels is maximum.

**Figure 9:**
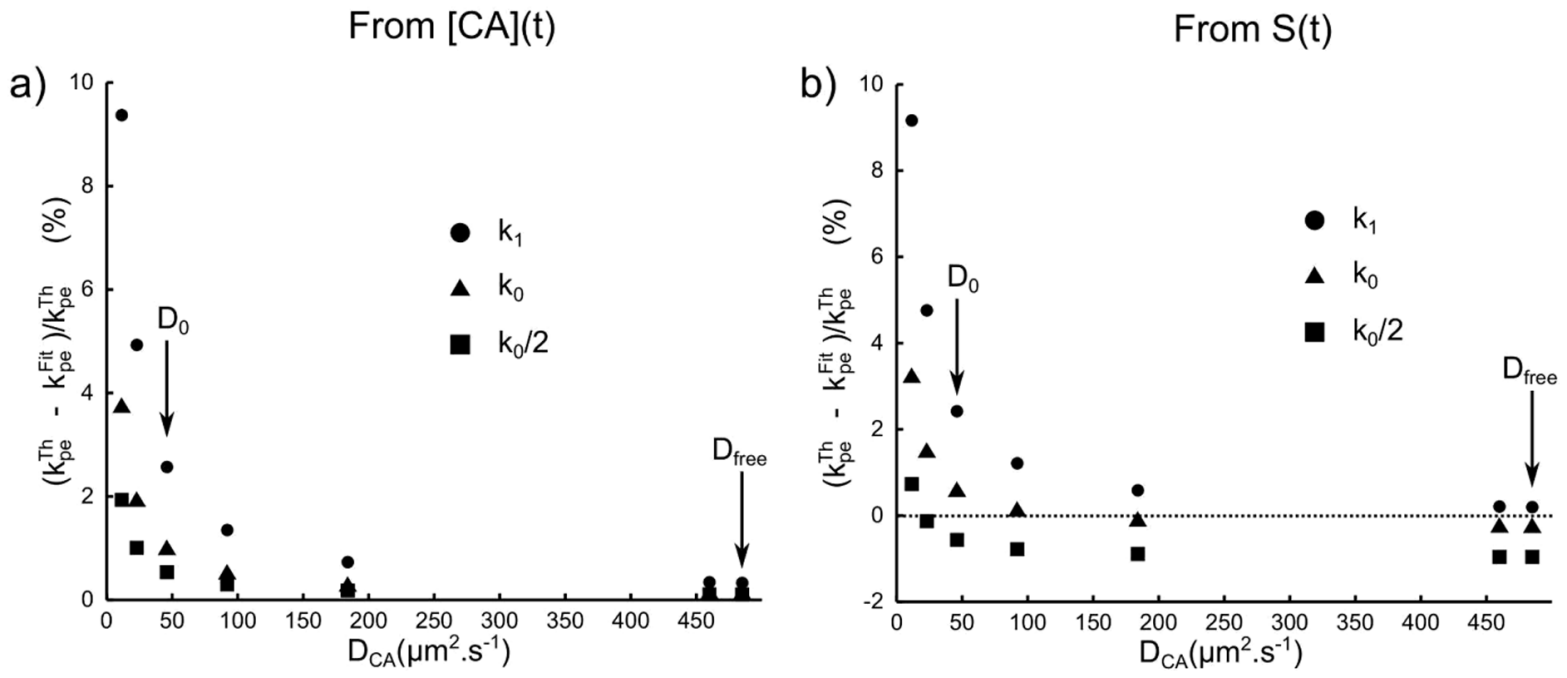
Error on the permeability estimate. When modeling the outputs of blocks b and c with Eq.[14] for various 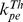 and 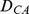 values: (a) Error on 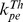 when modeling 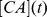. (b) Error on 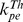 when modeling S(t) for 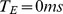 with Eqs.[16–17].

Fig. 9a presents the permeability estimates derived from 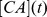. The higher the permeability, the larger the error on 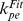. The faster the CA diffusion, the smaller the error on 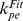. For the range of CA diffusion coefficients used in this simulation, the error never exceeded 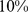. The highest error was obtained for the lowest 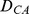 coefficient (Fig. 9a). This means that when the CA leaves slowly the vessel periphery, the vessel permeability is underestimated. As expected, this underestimation becomes smaller as 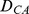 increases.

When the permeability estimates are derived from 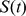 at 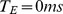, the behavior of the error on 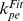 remains unchanged (Fig. 9b). For high 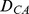 and low 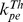, the errors become negative, in agreement with the result observed on Fig. 5b (slope smaller than one). The error on the permeability estimate remaines below 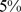. However, a difference between the permeability estimates obtained from 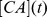 and 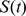 appears for high 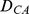 values : the error is independent of 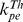 when one analyzes 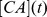 and becomes a function of 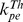 when one analyzes 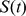. This difference may be ascribed to the 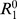 value used in Eq.[17] which assumes a slow exchange regime across the vessel wall (the effective 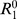 could not be computed since the water permeability was not controlled for in our model).

**Figure 10:**
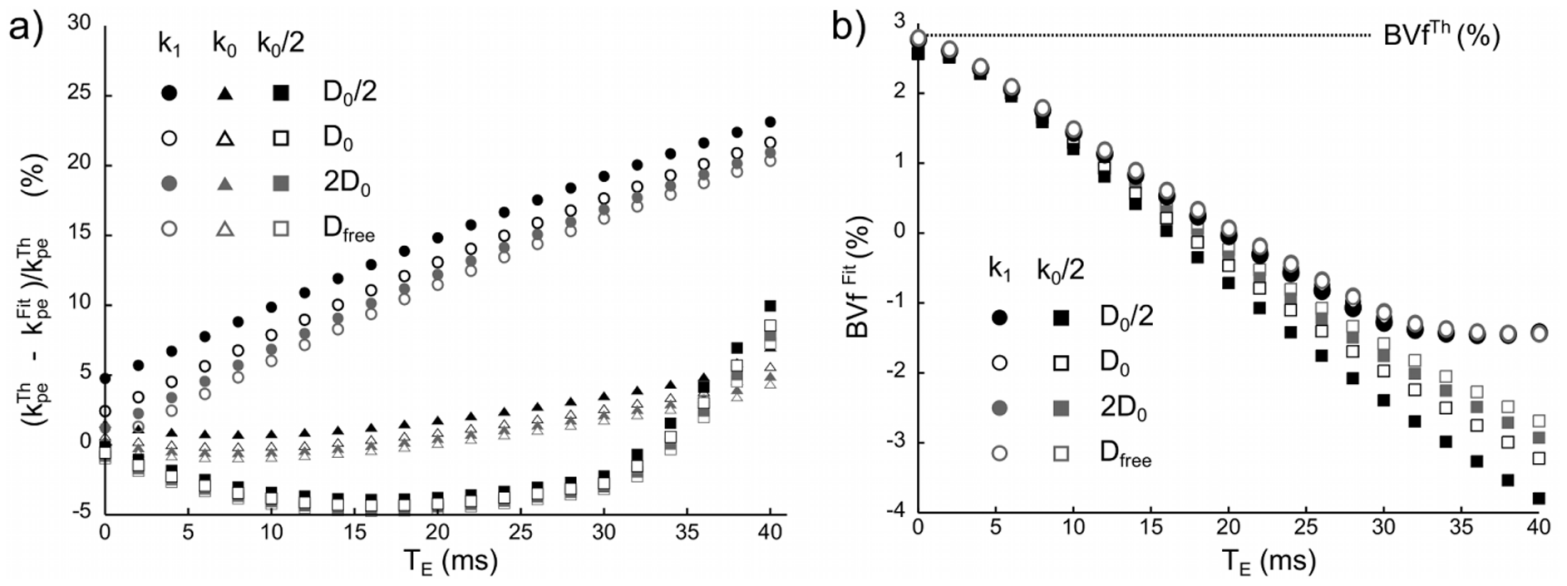
Impact of the echo time on the estimation of 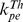 and 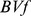. (a) Evolution of the error on the parameter 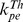 estimated from 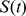 at different 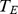 for various 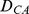 and various 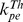. (b) Evolution of the parameter 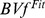 estimated from 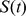 at different 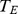, for various 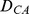 and for 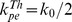 or 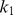 .

Fig. 10a shows the variation of the error on 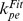 (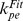 as a function of 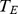 for different 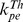 and 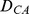). For short 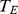, the error is minimum and the larger the CA diffusion, the lower the error as already observed in Fig. 9b. For 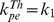, the error increases with 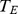. For 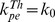 and 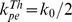 the evolution of the error is no longer proportional to 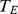 and ranges from 25% (for 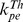) up to 200% (for 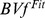). Fig. 10b shows 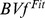 as a function of 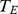 for different 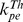 and 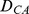. This error is the largest for long 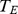. This deviation from the input 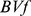 is ascribed to the post-processing: the 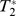 effects which are maximum at peak concentration are not taken into account in Eq.[17] (Fig. 8a). At this echo time, the data processing yields an erroneous estimate of 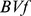 (Fig. 10b). For 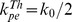, we note that 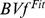 appears sensitive to 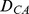 for long echo times.

The effect of the echo time on the permeability error is much stronger than that of 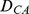. Going from 0 to 10 ms may increase the error from 2% to 8%. This impact is even more serious on the 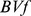 estimate: the same increase in echo time yields an underestimation of 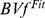 by a factor 2. This is likely related to the competition which occurs between the varying susceptibility gradients at the vessel wall and the increasing 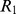 in the extravascular space. This balance depends on 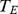 and on 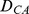.

### Versatility

#### Realistic microvasculature network

**Figure 11:**
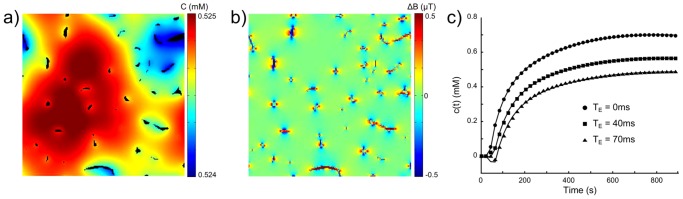
Example of the simulation with a vascular geometry extracted from in vivo microvascular microscopy. The simulation parameters are 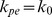 and 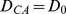. Concentration map 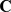 (a) and magnetic field perturbation 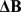 (b) are represented at the last simulation time point (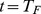). (c) Concentration profiles derived from the simulated MR signal using Eqs.[16–17] at 3 different 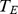. The black lines correspond to the fit obtained with the Toft model. Plane size 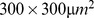.

To illustrate the potential of the proposed simulation tool, Fig. 11 shows the results obtained with a vasculature network extracted from a biological tissue. The geometry was acquired with a two-photon laser scanning microscope with 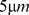 z-step [35]. Image size was 560
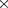
560 pixels with a field of view of 300
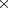
300 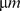. Morphological processing (erosion/dilatation) were used to fill holes in the vessels and to remove isolated pixels. A threshold was used for image segmentation (blood/tissue). A representation of the vascular network as the 2D binary lattice 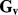 was eventually obtained. In Fig. 11, the blood vessels occupy 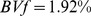 of the surface. The simulation was performed with 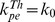 and 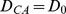. The lattices 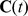 (Fig. 11a) and 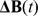 (Fig. 11b) are presented at 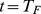. The concentration profiles derived from the simulated MR signal using Eqs.[16–17] are shown for 3 different 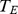 on Fig. 11c. The Toft model was eventually fitted to the data. The estimates were in agreement with the input values when 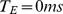 (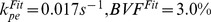) and biased for longer 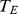 (at 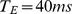, 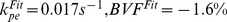).

#### Porosity of the extravascular space

**Figure 12:**
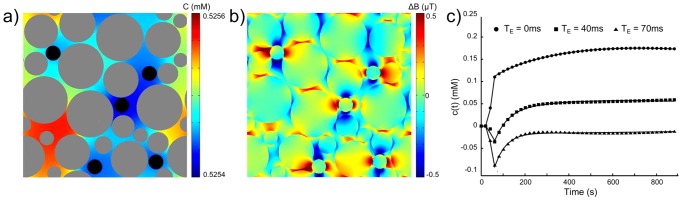
Example of the simulation with impermeable cells placed in the extravascular space. The simulation parameters are: 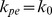 and 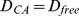. At 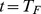 (a) Concentration map 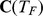 with vessels in black and cells in grey. (b) Magnetic field perturbation 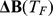. (c) Concentration profiles derived from the simulated MR signal and using Eqs.[16–17] at 3 different 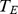. The black lines correspond to the fit obtained with the Toft model. Note the fluctuations in the concentration profiles obtained at long 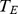. This can be ascribed to the additional magnetic field perturbations induced by the cell interfaces which balance the signal enhancement. Plane size 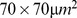.

Fig. 12 presents the results obtained with cells positioned in the extravascular space. Vessel geometry was the same as displayed on Fig. 3. The simulation was performed with 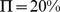, 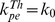 and 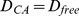. The lattices 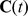 (Fig. 12a) and 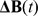 (Fig. 12b) are presented at 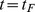. The concentration profiles derived from the simulated MR signal using Eqs.[16–17] are shown for 3 different 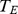 on Fig. 12c. The Toft model was also fitted to the corresponding data and the results were in agreement with previously described ones (at 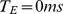, 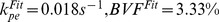) and biased for longer 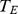 (at 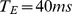, 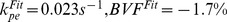)). The difference in the concentration scale is due to the reduced interstitial volume. For long echo times, the additional magnetic field perturbations that arose at the cell membranes reduce the enhancement effect and inflection points can be noticed in the concentration profiles (Fig. 12a at 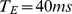 and 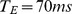).

## Discussion

In this study we proposed a tool to simulate a DCE-MRI experiment with intravenous CA injection and altered BBB. The simulation tool takes into account the blood flow, the CA extravasation via a controlled vessel wall permeability, the CA diffusion within the extravascular space, the water diffusion, the vessels and CA susceptibility effects together with the relaxivity effects. More realistic models for flow such as plug-flow or not well-mixed blood compartment are disregarded. Each important step of the simulation was validated and we discuss the deviation observed in the following. At various echo times, the impact of the CA diffusion on the permeability estimate was investigated in the limit of high flow. The versatility of the algorithm was finally demonstrated with geometries based on a realistic vascular network or space constrained by cells. The results obtained were in agreement with previously described in vivo experiments.

### Fourier based approaches

The simulation benefits from the extensive used of the FFT for the computation of the magnetic field perturbations, the diffusion of the CA and the diffusion of the water molecules. In addition to the gain in speed compared to classic convolution algorithms, we take advantage here of the intrinsic properties of the discrete Fourier transform: the spatial sampling yields a spatial periodization. Thereby, with the diffusion kernel approach, the CA which leaves the lattice on one edge comes back on the opposite edge, as if the lattice was surrounded by similar lattices. Consequently, our model takes into account the contribution of the CA movements due to diffusion and arriving from adjacent voxels [39]. Interestingly, this approach, which is also relevant in 3D, can be extended to any geometries with obstacles such as extravascular cells (Fig. 12). This property also applies to the magnetization carried by the water molecules, which freely diffuse within the voxel, and to the magnetic field computation. Note also that the Gibbs artifacts at the border were here avoided thanks to the periodization of the 2D vasculature. These artifacts usually arose at the border of 3D voxel simulation and compel to reject the spoiled outer volume of the 3D lattice for the signal computation.

### CA diffusion

The main asset for using the kernel approach to model diffusion is its computational efficiency. The diffusion process appeared however slightly slower than with the MC approach. This reduction is related to the use of the weighted lattice 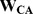: the amount of CA that diffuses from a pixel located within the interstitium to pixels located inside a vessel or a cell is sent back to it initial position. This differs from the MC approach where elastic collisions are considered. Thus, the CA diffusion modeled with the kernel approach is more hindered at the vicinity of vessels and cells than with the MC approach. From a physiological point of view, note that it has been proposed that diffusion near cells could be reduced beyond elastic collision due to electrostatic interactions with cell membranes [40], [41].

The kernel must also be narrow enough to avoid ‘jumps’. When the kernel width, 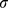, is larger than the characteristic obstacle size, 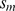, the CA diffusion becomes blind to obstacles. To avoid this behavior, one must observe 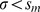. This ‘no-jump’ condition was respected in this study: 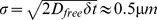 and 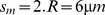. This condition may be fulfilled for a large range of 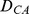 values and obstacle sizes by adapting 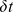 accordingly.

It is also worth noting that, by adding kurtosis or skewness terms to the kernel, the kernel approach offers the opportunity to model non-Gaussian diffusion and/or active transport.

### Magnetic field perturbations

The approach used in this study to compute the magnetic field perturbations is based on the Fourier transform of the magnetic susceptibility lattice [20], [21]. Most works employed the analytical form of the magnetic field perturbations generated by an infinite straight cylinder [23], [36], [37], [42], [43]. However, this latter technique, computationally efficient, fails at computing smooth susceptibility variations and more realistic vasculatures which vessel calibers, vessel densities and tortuosity may be significantly modified as in tumors [2], [24], [44]. Another approach, based on the perturbation produced by a single pixel convolved with a geometry lattice (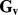 in our study) [38], has been proposed. This latter approach is adapted for arbitrary vessel geometry, but not for arbitrary susceptibility distribution.

By adapting the Fourier based approach to 2D, we decreased the computation time by about 400 times compared to the analytical approach. In 2D, the single vessel geometry yielded a 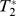 shorter than what was obtained with multiple vessels. Note that, due to the FFT, the single vessel geometry actually models a periodic vessel distribution. It thus appears that the regularity of the vessel arrangement has an impact on the eventual 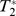 of the voxel. With a single vessel arrangement, the dipolar effect is spread uniformly over the plane and does not overlap with that of other vessels. Additionally, the distance between any pixel of the plane and a vessel is minimized. Thereby, one maximizes the dipolar effects of the vessel over every points of the lattice. 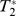 appears sensitive to the vessel arrangements (with constant 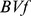), as observed on Fig. 7 where the standard deviation on 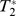 arises from ten different vessel arrangements. Note that this standard deviation is relatively modest (about 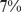), despite the use of only 5 vessels in each arrangement. Further studies are required to determine the optimal number of vessels per arrangement and the optimal number of arrangements to obtain reliable results in an optimized computation time.

Single or multi-vessel, 2D, geometries yielded shorter 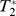 than 3D vascular geometries with comparable 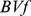 (Fig. 6). This difference comes from the fact that the magnetic field perturbation depends on the vessel orientation within 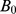. When all vessels are perpendicular to 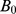, the magnetic field perturbation is maximized and the 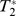 is reduced compared to what would be obtained with a random distribution of vessel orientations. Interestingly, we observed that averaging the signal from three orthogonal planes yielded 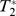 comparable to what was obtained with 3D approaches. One should keep in mind that this pseudo-3D approach requires the use of microvascular characteristics representative of the 3D distribution (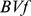, vessel diameter, vessel arrangements).

### Limitations

The main limitation of the 2D approach is related to the simulation of the flow within the voxel. Since only a plane is considered in our approach, one can not model plug-flow where CA concentration varies along the capillary bed. The study of the residue function is also harshly limited in our approach. The dispersion of the bolus brought by limited flow and the leakage can be modeled but the dispersion along the vascular tree can not be taken into account easily. With a sufficient number of vessels spread out within the plane, one way to overcome this limitation might be to define different residue functions for each vessel which would model the dispersion of the bolus at different length along the vascular paths. That is to take a section of a 3D voxel with mixture of arterioles, capillaries and venules as the one used in [45]. Note also that the well-mixedness assumption of the blood compartment may not stand in vivo.

As the expense of computational time, these two restrictions can be overcome by extending our approach to a 3D voxel. In that case, vessels are usually modeled by straight cylinders. One could also used 3D microvascular networks, such as the one recently studied by Guibert et al [46]. However, the periodic boundary condition does not stand any longer in 3D. One usually deals with this limitation by considering only the MR signal provided by an inner volume where water molecules have not undergoe harsh distortion of the magnetic field caused by discontinuity at the border. However, this can not be used with a reasonable voxel size in the scope of long DCE-MRI simulation where transport of the water and the CA must remain coherent for several minutes. Recently, an elegant solution to this problem has been considered in modeling the vascular network generated by a random walker moving with a significant inertia under periodic boundary conditions [47].

Another strong assumption of our model is related to the free diffusion of water molecules. The permeability of the membranes to water affects the contrast of the MR signal. Different regimes have been considered into the past [7] and means to measure water permeability at the vascular wall are still under development [48]. Based on the approach used for CA transport in this study, the water transport across membranes could be modeled. Thereby, transcytolemnal water exchange could be accounted for [49] at the expense of computational time.

The specific saturation-recovery sequence used in this study may appear very specific. In particular TR is longer that usual. This is due to the specific requirements of DCE-MRI experiments acquired with spirals readout [26]. This however may lead to peak saturation when estimating the AIF in vivo. Other MR sequences can be modeled with our approach such as spoiled GE or inversion-recovery sequences. RF spoiling can easily be implemented. For gradient spoiling, the gradient strength must be adapted to the lattice size so that the phase evolution of the water molecules remains coherent at the edges of the lattice. Such a sequence is provided with the source code but additional validation steps are required.

When applying the Tofts model for the analysis, an exact AIF was assumed. The AIF estimation is critical in DCE and is often considered as the main source of error in the permeability estimate. An alternate way is to derive the AIF from the MR signal provided by the blood compartment. This would be more similar to an in vivo situation and the AIF peak saturation effect could be investigated depending on the MR sequence used.

To be more practical the simulation should also incorporate a noise model for the signal. This was not included in the model since the aims was to disentangle the various contributions to the signal. Given the small impact of the CA diffusion on the permeability, it might be worthwhile to confirm if the effects described are observed in presence of noise.

The MR simulation strategies proposed in this study could also be associated to other physiological simulation environments which take into account more complex and realistic compartment models (for instance MMID4, Multiple indicator, Multiple path, Indicator Dilution 4 region model, National Simulation Resource, Department of Bioengineering, University of Washington, Seattle, WA, USA). Extensions of the algorithm to the fields of DSC-MRI or arterial spin labeling could also be considered.

This simulation source code is under the GNU GPL license and is available at http://neurosciences.ujf-grenoble.fr/equipe5.

## Conclusion

We proposed a versatile 2D simulation tool to model the MR signal in DCE-MRI experiments. We presented how we combined the compartment approach used for DCE-MRI analysis with the physical mechanisms involved in MR contrast. Additionally, we provided a means to efficiently simulate the diffusion of the CA in presence of impermeable compartments. While our results are consistent with in vivo results, some improvements and optimizations are still required. The variability of the MR signal across different sets of vascular networks must be studied (number of vessels and distribution in space). The model of the blood flow can be refined and the effect of the arising magnetic field perturbations due to extravascular cell interfaces and CA leakage requires further investigations.

Many other perfusion studies may be foreseen using the proposed approach. Different MRI sequences can be investigated. Different biophysical models used to analyze DCE experiments can be compared. Various techniques employed to correct for CA extravasation in DSC experiments can be evaluated. The shape of the AIF can also be optimized. These studies may be performed at all magnetic fields and for different tissue types, adjusting parameters such as cell porosity, cell magnetic susceptibility, vessel radius or density. These numerical approaches, in addition to providing a means to deepen our understanding of DCE-MRI, are extremely desirable from an ethical and a financial points of view.
