# Correction: A Simulation Tool for Dynamic Contrast Enhanced MRI

**DOI:** 10.1371/annotation/0263ccf5-239b-48d7-8880-5f4b6b709846

**Published:** 2013-09-09

**Authors:** Nicolas Adrien Pannetier, Clément Stéphan Debacker, Franck Mauconduit, Thomas Christen, Emmanuel Luc Barbier

In the PDF, second author Clément Stéphan Debacker is incorrectly indicated as being a senior author. In addition, Debacker was not correctly indicated in the PDF as having contributed equally to the work with first author Nicolas Adrien Pannetier. 

